# Supplementary material for: Inflammatory factors and risk of lung adenocarcinoma: a Mendelian randomization study mediated by blood metabolites
Source: Front Endocrinol (Lausanne). 2024 Aug 27;15:1446863. doi: 10.3389/fendo.2024.1446863 (PMC11384989; doi:10.3389/fendo.2024.1446863)
Supplement: Supplementary file 1 [file DataSheet1.zip › supplementary files/S2/GCST90200922/forest.pdf]

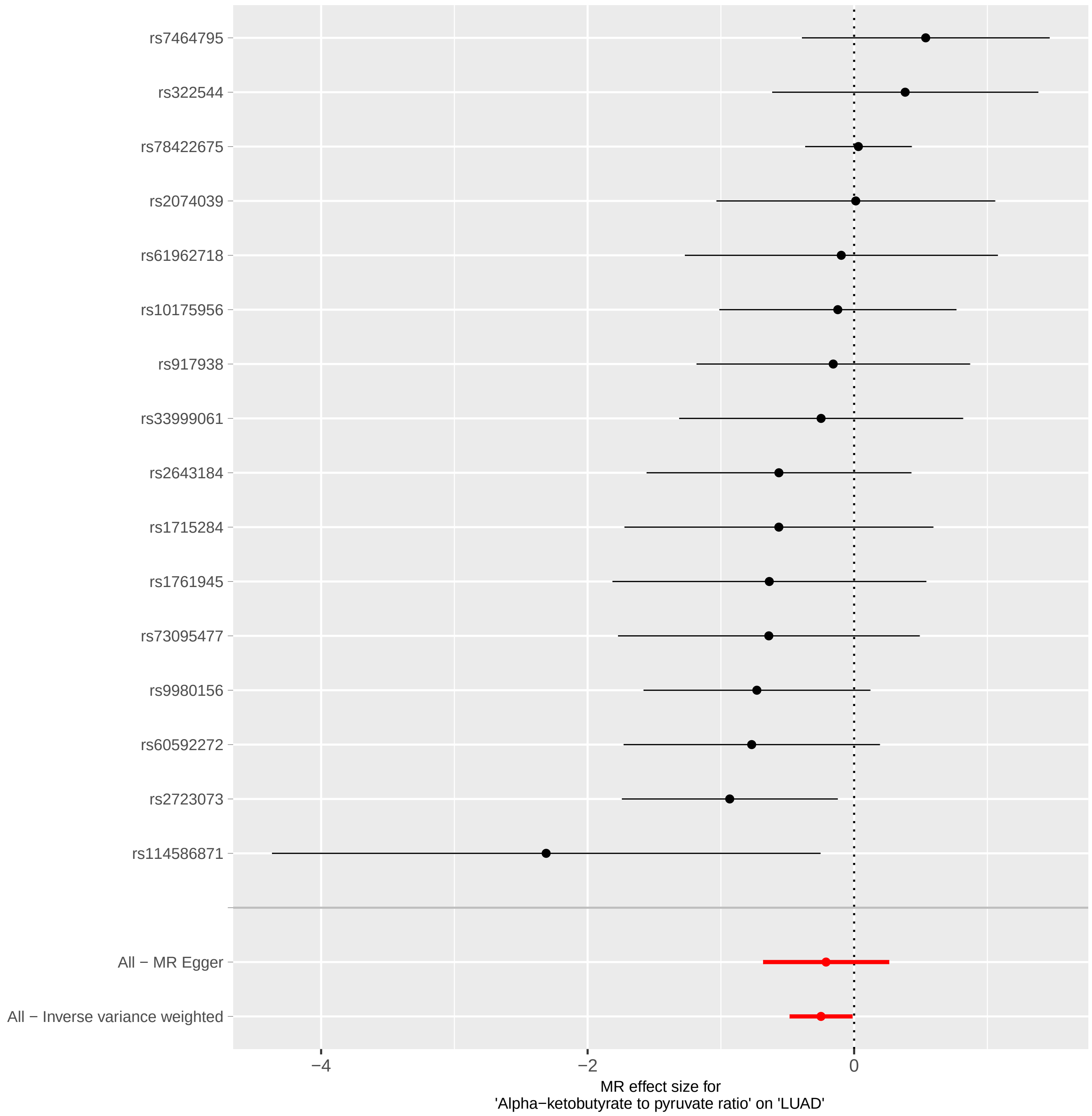
rs7464795
rs322544
rs78422675
rs2074039
rs61962718
rs10175956
rs917938
rs33999061
rs2643184
rs1715284
rs1761945
rs73095477
rs9980156
rs60592272
rs2723073
rs114586871
All − MR Egger
All − Inverse variance weighted
−4
−2
0
MR effect size for
'Alpha−ketobutyrate to pyruvate ratio' on 'LUAD'
